# Core competencies in occupational medicine from the perspective of the different fields of professional activity

**DOI:** 10.47626/1679-4435-2023-1168

**Published:** 2024-11-14

**Authors:** Rodolfo Miguel Alves Carniato, Marcia Cristina Bandini, Sérgio Roberto de Lucca, Valmir Antonio Zulian de Azevedo

**Affiliations:** Área de Saúde do Trabalhador, Departamento de Saúde Coletiva, Faculdade de Ciências Médicas, Universidade Estadual de Campinas, Campinas, SP, Brazil

**Keywords:** occupational medicine, professional competence, job market, medicina do trabalho, competência profissional, mercado de trabalho

## Abstract

Occupational physicians are expected to be ethical, technical, and humanistic professionals, working to promote and prevent work-related illnesses and to preserve worker’s health. The Competências Essenciais Requeridas para o Exercício da Medicina do Trabalho (Core Competencies Required for the Practice of Occupational Medicine) guidelines should be a reference in their professional practice. This article aimed to analyze whether the domains currently proposed in the competencies of the specialty are reflected in the professional practice of those interviewed. This is a qualitative study including 12 interviews with occupational physicians in different fields. The narratives were recorded and the content was categorized according to the competencies matrix of the specialty. The analysis of the speeches showed that all the participants addressed the five domains of the Competências Essenciais Requeridas para o Exercício da Medicina do Trabalho. The interviewees stressed the positive and critical aspects of training and professional practice and the need for constant learning updates. According to the interviewees, the competencies are suitable for the various fields of work in occupational medicine. Special attention should be paid to ethical and moral dilemmas, which are a permanent challenge for occupational physicians who deal with conflicting interests between employers and workers on a daily basis.

## Abstract (pt)

Espera-se que o médico do trabalho seja um profissional ético, técnico e humanista e atue nas ações de promoção e prevenção das doenças e agravos relacionados e de preservação da saúde dos trabalhadores sob sua responsabilidade. As diretrizes de Competências Essenciais Requeridas para o Exercício da Medicina do Trabalho devem ser referência na atuação profissional. O objetivo deste artigo foi analisar se os domínios atualmente propostos nas Competências da especialidade se refletem na prática profissional dos entrevistados. Tratou-se de estudo qualitativo com 12 depoimentos de médicos do trabalho de diferentes campos de atuação. As narrativas foram gravadas, e o conteúdo foi categorizado de acordo com a matriz de Competências da especialidade. A análise dos discursos demonstrou que todos os participantes abordaram os cinco domínios de Competências Essenciais Requeridas para o Exercício da Medicina do Trabalho. Os entrevistados apontaram aspectos positivos e críticos necessários na formação e atuação na prática profissional e a necessidade de constante atualização de conhecimentos. De acordo com os depoimentos, as Competências apresentam-se adequadas para diversos campos de atuação em medicina do trabalho. Atenção especial deve ser dada aos dilemas éticos e morais que constituem um desafio permanente para médicos de trabalho que lidam cotidianamente com interesses antagônicos entre empregadores e trabalhadores.

## INTRODUCTION

Occupational medicine is the medical specialty that deals with the relationship between workers and their work, not only aiming to prevent accidents and occupational diseases, but also to promote health and quality of life. It seeks to ensure or facilitate continuous improvement in the physical and mental health of individuals and groups of workers, and to promote healthy interaction among individuals and their social and work environments. It is built on two pillars: clinical and public health. It focuses on preventing and caring for workers who are victims of work-related accidents, diseases or disabilities, and on promoting occupational health, well-being and productivity for workers, their families, and their community.^1^

The scope of occupational medicine is broad, going beyond the traditional scope of medical practice.^2^ Schematically, it can be described as being preferably practiced:

- In companies, public or private, in the Serviços Especializados de Engenharia de Segurança e de Medicina do Trabalho (Sesmt, Specialized Services of Safety Engineering and Occupational Medicine), as an employee or as a service provider for the preparation of the Programa de Controle Médico de Saúde Ocupacional (PCMSO, Occupational Health Medical Control Program), among others;
- In standardization and inspection of Occupational Health and Safety (OHS) conditions developed by the Brazil’s Ministry of Labor;
- In public health care networks and in the development of worker health actions;
- Trade union advice on workers’ health, at workers’ and employers’ organizations;
- In the Perícia Médica Federal (Brazil’s Medical Examination System), at the Instituto Nacional de Seguro Social (INSS, Brazil’s National Social Security Institute) or other institutions;
- In the Judicial System as a court expert in labor cases, civil suits and actions by the public prosecutor’s office or the Ministério Público do Trabalho (MPT, Labor Prosecutor’s Office);
- In teaching and professional training;
- In studies in the field of health and labor relations; and
- In private consultancy in the field of OHS.

The Competências Essenciais Requeridas para o Exercício da Medicina do Trabalho (Core Competencies Required for the Practice of Occupational Medicine) have to be developed to ensure adequate professional performance in as many fields as possible. Le Boterf^3^ defines competency as knowing how to act in a responsible and recognized way, which involves mobilizing, integrating, and transferring knowledge, resources, and competencies that add economic value to the organization and social value to the individual. It is therefore not an innate ability, but one that is developed through motivation. The development of competencies is a dynamic process, which is constantly being reformulated according to new scenarios, changes in context, and society’s expectations.

The first version of the Competências Essenciais Requeridas para o Exercício da Medicina do Trabalho dates from 2003^4^ and is the recognition of medical specialties established by the Conselho Federal de Medicina (CFM, Federal Council of Medicine), the Associação Médica Brasileira (AMB, Brazilian Medical Association), and the Comissão Nacional de Residência Médica (CNRM, Brazil’s National Commission of Medical Residency).^5^ This recognition meant the beginning of the training of occupational physicians through direct access medical residency, and the Competências Essenciais Requeridas para o Exercício da Medicina do Trabalho developed with the support of the Associação Nacional de Medicina do Trabalho (ANAMT, Brazil National Association of Occupational Medicine) became a reference for the development of new residency programs. In 2016, the Competências Essenciais Requeridas para o Exercício da Medicina do Trabalho were updated using the Delphi methodology.^6^ In 2018, this process culminated in the publication in three languages of the current version of the Competências Essenciais Requeridas para o Exercício da Medicina do Trabalho.^7^ It contains the five competency domains, which are:

1. The competency of “moral judgment or professionalism,” which is the professional’s ability, when faced with concrete situations, to reflect using critical reason, knowledge, and values to decide on the most appropriate practices and conduct, considering the workers’ right to health and life;
2. “Comprehensive care for workers’ health,” at an individual and a collective level, through health promotion and protection, surveillance and assistance, including physical and professional rehabilitation;
3. The competencies related to the “study of work,” including the assessment, analysis, and intervention in risk situations, present or potential, for the health and physical and mental integrity of the worker. This involves knowing the work processes, their technical and sociopolitical dimensions, recognizing the present or potential risks to workers’ health and well-being, indicating and analysing specialized studies to guide decisions on changes to the processes and strategies for protecting workers;
4. The “formulation and implementation of policies and management of workers’ health,” at an individual and collective level, is a competency valued by employers, especially in the leading sectors of the economy, and involves the integrated management of Health, Safety at Work and the Environment (HSE); and
5. The “transversal competencies” are those that cut across all the other domains, involving: a) knowledge and appropriate use of legislation applied to OHS; b) mastery of verbal and nonverbal communication skills and interpersonal relationships, based on dialogue and empathy, aiming at ensuring the right to information, the dissemination and improvement of understanding of issues related to health and the integration of people at the workplace; c) knowledge management aimed at the ongoing improvement of professional practice; and d) the skill to work in a team, including the exercise of leadership and conflict mediation.

Finally, it is worth noting that the CNRM incorporated the Competências Essenciais Requeridas para o Exercício da Medicina do Trabalho (2018) into the specialty’s competency matrix, which guides residency programs and is considered the gold standard of medical training.^8^ This study is based on the context of the diversity of professional fields and the need for structured training in competencies, with a requirement for continuing education.

Based on the analysis of 12 interviews with occupational physicians in different areas of practice, the study analyzed the discourses in the light of the Competências Essenciais Requeridas para o Exercício da Medicina do Trabalho, seeking to identify whether the domains currently proposed are reflected in the professional practice of the interviewees.

## METHODS

This is a qualitative study using discourse analysis of 12 recorded interviews, whose empirical and documentary material was obtained through a teaching activity in the occupational medicine residency program at the Faculdade de Ciências Médicas (School of Medical Sciences) da Universidade Estadual de Campinas (UNICAMP, State University of Campinas). The interviewees consented to the videos being broadcast on the university’s YouTube channel.

Specialists in occupational medicine with outstanding experience in the following fields were invited: (1) a private company manager; (2) a medical director of a group medicine company providing occupational medicine services; (3) a medical advisor to a workers’ union; (4) a medical director of surveillance and attention to workers’ health, at state level; (5) a federal medical expert; (6) a labor inspector; (7) a medical expert for the MPT; (8) a doctor for the Tribunal Regional do Trabalho (TRT, Regional Labor Court); (9) a founding partner of a company providing services in occupational medicine; (10) a technical director for the Serviço Social da Indústria (SESI, Social Service for Industry); (11) a representative of an association of occupational health and safety companies; and (12) a lecturer at a public university.

The interviews were conducted based on two trigger questions: “What has your professional career been like?” and “What is your everyday professional routine like?”, in a free format, with the possibility of questions from the residents participating in the educational activity. The interviews lasted 50 minutes and were conducted during the pandemic period, with restrictions on face-to-face activities, over the course of 3 months. The interviewees were identified by the letter “E” followed by the number of their field of work. The recordings were transcribed, and the narratives were categorized according to the five domains of Competências Essenciais Requeridas para o Exercício da Medicina do Trabalho (2018).

## RESULTS

The discourse analysis showed that all five domains of the Competências Essenciais Requeridas para o Exercício da Medicina do Trabalho were addressed by all the interviewees. The most relevant statements are transcribed below, based on the domain.

The “moral judgment or professionalism” domain was addressed by all the interviewees, with reports of daily challenges and conflicts, of which the following stand out:

“(...) *the most important thing is to know how far we’re going to go, whether I accept it or not (...) those who have the courage to say ‘I won’t do it’ and ‘this is no place for me to work’ are the ones who are better placed in the market because the company knows how to differentiate one [employee] from others and they also want to be confident in the people they hire (...) if I say I’m going to issue a Comunicação de Acidente de Trabalho (CAT, Work Accident Report) and they tell me they won’t accept it, this company is not for me* (...).” E3

“(...) *centered on the ethical issue, this is the occupational physician’s biggest challenge because they deal on a tightrope between the very powerful interests of the productive sector and the interests of workers’ health and lives, so the development of moral competency for us comes somewhat from our upbringing, from home, from family, but it can also be learned, especially when it comes to responding to conflicts* (...).” E12

“(...) *I know that some companies are impossible to work for, you can’t stay because the ethical issue can’t be dealt with”* and adds *“I was invited to work for a very large company, and they said that here in the company you don’t issue a CAT, if you have an occupational illness, let the union issue it, let the worker look for it, because here you’re not going to screw over the company; I said that, unfortunately... you can’t stay in a company like that* (...).” E7

“(...) *the worker has been diagnosed with cancer, is about to start a long course of chemotherapy, radiotherapy, etc.; the cost of the health insurance goes up, (...) then the doctor says if they’re able here, they’re working, I can dismiss them and it’s not an occupational disease, this is unethical and characterizes a discriminatory dismissal in the labor courts and then this guy is going to be reinstated, with good reason* (...).” E10

“(...) *yes, I have been paid, I believe that all experts must have been paid at some point in their lives, but it’s veiled, right, the person doesn’t come up to you and say: ‘doctor, how much money do you want to give this favorable/unfavorable report?’ Things are veiled. Operation Hipócritas, as far as I know, the business had already gone off the rails, I know of situations that I can’t tell you... but it seems to me that it was quite clear that the experts were saying ‘if you pay me an amount of money, you’ll have a report’... there are both sides, there’s the evil expert who gets paid for not providing a causal nexus and gets paid by the company on the side, and there’s the evil expert who provides a causal nexus to everything because they’re not thinking about the worker’s advantage and getting their fee.*” E10

“(...) *in order to be a good doctor, a good professional, a good technician in the Sistema Único de Saúde (SUS, Brazil’s Unified Health System), wherever we are, it’s important that we work with what we believe in, which are our principles...*” E4

In terms of “comprehensive care for workers’ health,” at an individual and a collective level, the interviewees reported on the need to go beyond legal compliance, such as preparing the PCMSO, among others:

“(...) *there was no point in me just looking after occupational health and not looking after people’s lifestyle because employees are a whole being, not just what they are at work, there’s no point in me just looking at occupational risks. As an occupational physician, you have to take a holistic view of the picture. It wasn’t just about conducting the PCMSO in relation to risks, but also the PCMSO in relation to their lifestyle* (...).” E1

“(...) *I love occupational medicine, I think it’s a very rich, wonderful field, it’s what I wanted to do, I like preventing illnesses and fostering health.*” E9

“(...) *we have a huge field of action, we make a synthesis in our daily lives between the individual and the collective, we take care of these groups indiscriminately, we work from the biological to the ultracellular and with the most macro social that can exist and this is our fascination, we aggregate at the root of our specialty everything that comes from the human sciences, the arts, cutting-edge technology, and these are the requirements to be a good occupational physician.*” E12

In the competencies relating to “study of work,” references to the need to defend occupational health as an investment, not an expense, and the importance of being prepared for inspections stand out:

“(...) *you have to prove conclusively how much money will be saved if you invest in safety, work-related accidents are prevented, and this is the calculation we make for the business owner, investments in safety can’t be seen as a cost (...) if there’s an accident in the company, you’ll spend ten times more than the investment in safety* (...).” E2

“(...) *one of the most important things is to understand the subject, if I’m going to talk to someone at a bank, I can know everything about occupational hygiene, but I won’t be able to talk to them, negotiate with them, because occupational hygiene isn’t the bank’s métier, these are psychosocial issues, I’ll have to understand occupational sociology, other subjects, other topics that are more specific to that particular work situation* (...).” E7

“(...) *labor inspection, which is conducted by tax auditors, we call it federal inspection or labor inspection, and the SUS, based on the Constitution and Law 8080, which is now 30 years old, which is from 1990, establishes that the SUS is also responsible for monitoring the environment, including the work environment... These are activities that in practice include going to the workplace, checking the conditions, checking what is there, but with some particularities, differences from the point of view of the applicability of the legislation and from the point of view of fines, for example. Labor inspection applies the regulatory norms much more particularly, there are notices, fines for failure to comply with these norms, much more applied to permanent workers...*” E6

The “formulation and implementation of policies and management of workers’ health” at an individual and collective level remains a challenge for professionals, especially in the context of the pandemic or situations of harassment:

“(...) *the pandemic has provided physicians at work with this opportunity to gain ground and be valued within the company, how they manage the chronically ill who are on leave, the population working from home, they need to understand the family of that employee who has a child in the risk group or lives with a mother in the risk group, map this population within the company* (...).” E11

“(...) *we have the recognition through the professional technical nexus that has a relationship with the list of work-related diseases of Brazil of 1999, and that through these lists you have a relationship of risk exposures at work and the relationship with some incapacitating disease, or we evaluate by the epidemiological question that is the technical epidemiological social security nexus that is a study of the diseases that most affect certain groups of workers and therefore, through epidemiology, we characterize this relationship of the disease with work* (...).” E5

“(...) *a person gets sick and leaves, we see that long periods of leave are very difficult to come back from, the person loses a bit of their identity in the workplace, there’s a huge stigma towards people who stay away for a long time and it’s not uncommon for us to find a specific harassment, but at least nuances of specific harassment in this person’s illness* (...).” E8

“(...) *what is written in the norm, what is prescribed often fails to address reality, the real world, the dynamism of concrete working conditions... this is a learning experience.*” E4

Finally, the acquisition of “transversal competencies” is a concern for many professionals:

“(...) *we have to have management software to compile information because if we don’t have management software, theoretically how are we going to manage at a Brazilian level?* (...).” E2

“(...) *An MBA is another course that makes a difference, I don’t regret having taken an MBA, I learned a lot in practice, and it makes a big difference when companies hire people today, we end up getting many applications for medical managers, coordinating medical managers who have had experience or some kind of management course* (...).” E11

“(...) *understanding that the occupational physician has to be currently open to activities outside of occupational medicine, strictly as in the operational, regulatory area, yes, you have to build relationships within the company, have a different attitude when you go to an executive meeting, you have to think a little outside the box of what’s on the agenda* (...).” E11

“(...) *I attend groups, I have contact with the judiciary all over the country and today the occupational physician makes a big difference within the judiciary, we see the difficulty that other colleagues have who don’t have the training to respond to the demands that are placed on them today... “Sometimes the dialog with the authorities isn’t very smooth, especially the judge, I’m not talking about all of them, they don’t accept a lot of suggestions, they don’t like you intervening in their work, they don’t take criticism very well, sometimes the things that need to be changed, small things, we can’t change. Today we’re flooded with complaints of bullying, some we manage to deal with, others we can’t...*” E9

## DISCUSSION

This study shows that the Competências Essenciais Requeridas para o Exercício da Medicina do Trabalho (2018) are suitable for professional practice in different fields of the specialty, since all the interviewees mentioned the five domains in their narratives. All the interviewees reported dilemmas and conflicts in their professional practice. Thus, moral judgment or professionalism is central to the practice of occupational medicine, since the professional is crossed by conflicting and antagonistic interests, such as the relationship between workers and employers; ethical issues, uncertainties of technical knowledge; among other ethical and moral dilemmas.

The interviewees agreed that the occupational physician is faced with a difficult mission in the face of conflicts of interest between employers and employees, in which a professional ethical attitude and moral judgment are the only possible way forward. According to Leonardo Boff,^9^ morality is a set of customs and values of a given society or group of people; therefore, acting morally does not necessarily mean that the action was taken ethically. However, when we talk about ethics, we mean the common good, which can be defined as access for all to basic goods and the right to recognition, respect, and peaceful coexistence in solidarity.

The difficulty of acting morally and ethically is also mentioned in Vázquez^10^:

it will be useless to turn to ethics hoping to find in it a rule of action for each concrete situation. Ethics will be able to tell you, in general, what norm-oriented behavior is, or what the end - the good - aimed at by moral behavior consists of, which is part of the procedure of the concrete individual or that of everyone. The problem of what to do in each concrete situation is a practical-moral problem and not a theoretical-ethical one.

In Beauchamp & Childress’s^11^ Principlism theory, the principles of respect for autonomy, beneficence, nonmaleficence and justice should be observed. Autonomy and nonmaleficence are also included in the Hippocratic oath^12^:

I will apply regimens and treatments according to my best discretion and conscience, never to cause harm or evil to anyone, even at the behest of anyone, and I will keep secret whatever I see, hear, or come to know in the practice of medicine [...] that need not be divulged.

These should be the ethical imperatives of the occupational physician.

The field of Comprehensive Health Care, which all participants mentioned, shows the relevance of the occupational physician as an actor in promoting health in the workplace. In addition to the individual and collective benefits for workers’ health, the interviewees reported that the management of corporate health insurance can benefit from a broader line of care, with lower rates of absenteeism and presenteeism.^13,14^ Thus, the occupational physician is a key player in the promotion of health in the workplace. The occupational physician can therefore act as a gatekeeper for supplementary health, similar to the family and community doctor in Primary Health Care (PHC), and based on the principles of health promotion, with emphasis on the fact that “the social organization of work should contribute to the constitution of a healthier society” and that “health promotion generates living and working conditions that are safe, stimulating, satisfying and enjoyable” (Ottawa Charter^15^).

As work is a central component of the social determinants of health, which include economic, social, ethnic/racial, cultural, behavioral, and psychological factors,^16^ in which working conditions figure prominently in the Dahlgren & Whitehead^17^ model, the occupational physician can play an important role in promoting individual and collective health. This requires going beyond simply controlling occupational risks and becoming a protagonist in protecting workers’ health.

As for the study of work, this responsibility extends to all doctors, according to the Code of Medical Ethics^18^ which, in Chapter I, Item XII, states that “the doctor shall strive for the best adaptation of work to the human being, for the elimination and control of health risks inherent in work activities.” Since it is the duty of all doctors, occupational doctors have an even greater responsibility, due to the nature of their specialty, to dedicate their time to studying the conditions, environments, processes and organization of work, providing protection and prevention guidelines for workers and employers.

There is an increasing demand for the development of competencies beyond medicine when it comes to formulating and implementing policies and managing workers’ health. Examples include the growing concern with issues related to Environmental, Social and Corporate Governance (ESG).^19^ With their competencies fully developed, occupational physicians can contribute to the “E - Environment,” helping to reduce waste from occupational health services; to the “S - Social,” promoting diversity in the workplace; and to the “G - Corporate Governance,” fighting discrimination and harassment and raising workers’ awareness of issues such as mental health and work.

Finally, transversal competencies are increasingly required in the practice of occupational medicine, with the need for continuing education and permanent updating on labor, social security, and health legislation. Communication and management skills, verbal and nonverbal language, and conflict mediation are and will continue to be increasingly required. It is therefore necessary for residency programs in occupational medicine to be reinvented and to propose new training alternatives. In addition, complementary training is and will be increasingly important for occupational physicians or, more correctly, for all physicians in Brazil.

## FINAL CONSIDERATIONS

Although this is an empirical analysis, based on the individual reports of occupational physicians, this study demonstrates the relevance of the Competências Essenciais Requeridas para o Exercício da Medicina do Trabalho, whether in planning medical residency programs or proposing continuing education programs. These competencies are suitable for different fields of work in occupational medicine. However, it is known that it is impossible to cover all the competencies in the 2 years of medical residency, and it is therefore necessary for complementary training programs to be developed for these professionals.

The ethical and moral dilemmas that constitute a permanent challenge for occupational physicians who deal with conflicting interests between employers and workers on a daily basis should be given special attention. In addition to formal theoretical training, it is necessary to propose more effective ways of providing moral and ethical training to support the professional practice of this medical specialty.
